# Why some BCI should still be called BMI

**DOI:** 10.1038/s41467-024-50603-7

**Published:** 2024-07-23

**Authors:** Ulrich G. Hofmann, Thomas Stieglitz

**Affiliations:** 1 Section for Neuroelectronic Systems, Department of Neurosurgery, Medical Center University of Freiburg and Medical Faculty, University of Freiburg, Freiburg, Germany; 2 BrainLinks-BrainTools, Freiburg, Germany; 3 Laboratory for Biomedical Microtechnology, Department of Microsystems Engineering-IMTEK, University of Freiburg, Freiburg, Germany

**Keywords:** Translational research, Biomedical engineering

## Abstract

Neurotechnology is becoming an integral part of clinical practice. In this Comment, the authors advocate for more scrutiny and attention when describing new neurotechnologies with particular attention to the surgical risks involved and the invasiveness.

Neuroengineering has reached new heights and promises to bring the next revolution in treating human ailments. After decades of academic incubation and technological ripening, the last years witnessed an explosion of success stories, and a number of clinical trials, reporting on the implantation of devices capable of mediating the connection between a human brain and a machine. Such devices build upon, and complement, neurotechnologies already established in clinical practice, such as deep-brain stimulation and cochlear implants, and hold potential for individuals with previously untreatable conditions, as well as for technology enthusiasts and investors alike.

It is important, however, to exercise caution and consider that not every innovation will seamlessly integrate into clinical procedures. Regulatory boards, such as the US Food and Drug Administration (FDA) and the European Notified Bodies, play a pivotal role in assessing the risk levels associated with these technologies and their deployment. These agencies meticulously assess and categorize medical devices based on the associated risks: for instance, any implanted device in direct contact with brain tissue, such as deep-brain stimulators or intracortical recording implants, is evaluated according to the highest risk group III, whereas a device placed on the scalp will be assessed according to the lowest risk class I. Consequently, devices categorized under lower-risk classes stand a greater chance of certification, akin to contemporary smartwatches.

Very much to our despair, and possibly offering a disservice to the public, most of the fruits of neuroengineering are nowadays indiscriminately dubbed “brain-computer interfaces” (BCIs), an umbrella terminology that obscures the risks associated with the surgical intervention they require. This is unfortunate as, up till the early 2000s, the term BCI was mainly reserved for non-invasive brain recordings^1,2^. Their invasive and intracortical counterparts, instead, were commonly referred to as “brain-machine interfaces” (BMIs)^3^. These terms were rarely used interchangeably and thus we advocate here—lacking a stronger and more resounding word pair—to return to the proven and unambiguous terms “BCI” and “BMI”. It must be acknowledged that the distinction was already blurring when the invasive Utah Silicon array was called a BCI in one of its inventor’s articles^4^. The distinction was ultimately shattered in 2013 when one of the first groups that reported results on human participants called their implanted system a BCI^5^.

In this context, and given the growing public interest in neurotechnology, we feel the urge to encourage the community to clarify these differences already in their denominations and motivate researchers, companies, and investors to be aware and meticulous about them. In the same spirit, last February, the BCI Society called upon the community to seek a more accurate definition of BCI (https://bcisociety.org/bci-definition/).

We do not believe the discussion about whether the acquired data remains “internalized” to drive tissue stimulation or is extracted to drive an external artifact will clear the fog. The main factor that should determine the definition of a brain implant has a more pragmatic nature: the safety during implantation and operation. Whether the device is placed in the blood vessels (intravasal), under the skin (subdermal), on top of the brain (epidural), or within the cortex (intracortical), it has to meet basic requirements concerning biocompatibility, sterilization, and longevity. Moreover, going beyond mere linguistic aspects and surgical considerations, additional key features set intracortical and non-invasive brain interfaces apart.

Another reason to distinguish between implantable BMI and non-invasive BCI is their signal transfer performance. Skin and skull represent a temporal filter that allows the passing of low frequencies up to 100 Hz only and delivers a low spatial discrimination. Implantable systems (BMI) bypass this filter, thus granting the acquisition of higher frequency components up to 100 kHz like spiking activity (from multiple or even single nerve cells). This bears important consequences for the information each signal channel can contribute to the medical purpose: According to Shannon’s noisy-channel coding theorem, the channel capacity is limited by the signal-to-noise level and the carrier frequency’s bandwidth. Since both signal sources are notoriously noisy, implantable devices have an edge in terms of possible information transfer rate and response times.

Moreover, recent histological and transcriptomics analysis revealed that implantation in brain tissue may lead to undesirable side-effects not limited to the well-known gliotic brain response^6^, but a possible persistent activation of the innate immune system and proto-oncogenes (like JUN, PER1, KDR)^7^. This appears to be a risk not yet addressed by the tests usually carried out for certifications and thus poorly addressed by the companies and startups that propose groundbreaking novel treatments. Further evidence to delineate the medical consequences of our observations is needed. Until then, we deem it inappropriate to subsume two distinct technologies under one “harmless” terminology, thereby obfuscating fundamental differences in their nature and their effects.

In conclusion, we encourage the community to scrutinize the ambiguity of the term BCI and to choose accurate wording and acronyms when proposing new technologies and devices. In the absence of immediate alternatives and a common consensus, we advocate to return to the classic denominations “BCI” and “BMI” to delineate at first sight the invasiveness, data throughput, and neuroinflammatory consequences of the neurotechnology at hand. Alternatively, the term “*i*BCI” might become defensible ground and quickly convey the idea of an invasive, intracortical, and implantable device. Science communicators, however, will need to use utmost diligence not to lose the innocent “i” when it is urgently needed by the public.

We believe these simple considerations will go a long way and effectively contribute to an honest and transparent conversation within the academic, clinical, and investor community. Ultimately, we hope this will raise the public’s trust in neurotech in the long run as well as increase the awareness of the end-users by offering, from the very first contact, a more informed way to decide which technological promise they could put their hopes on.
